# Dynamic Immune/Inflammation Precision Medicine: The Good and the Bad Inflammation in Infection and Cancer

**DOI:** 10.3389/fimmu.2021.595722

**Published:** 2021-02-23

**Authors:** Jean-François Rossi, Zhao Yang Lu, Cesare Massart, Kalle Levon

**Affiliations:** ^1^ Hématologie-Immunothérapie, Institut du Cancer Avignon-Provence, Sainte Catherine, Avignon, France; ^2^ Faculté de médecine Montpellier, Université de Montpellier, Montpellier, France; ^3^ Unité de Thérapie Cellulaire, CHU Montpellier Saint-Eloi, Montpellier, France; ^4^ E-SANA, Orsay, France; ^5^ New York University (NYU) Tandon School of Engineering, Six Metrotech Center, Brooklyn, NY, United States

**Keywords:** inflammation, infection, cancer, precision medicine, immune therapy

## Abstract

Normal or “good” inflammation process starts from a local cellular response against injury or any infectious agent, with the activation of neutrophils, macrophages, Langerhans cells, dendritic cells, and innate immune cells. Cytokines and chemokines are produced to amplify the local inflammatory process followed by the migration of immune cells to the regional lymph nodes where adaptive immune response is initiated. Systemic inflammation enhances the biological response to mobilize additional cells from central and peripheral immune/hematopoietic system. Local mechanisms to limit inflammation are initiated and lead to healing. During the normal inflammatory process, there is a balance between the production of inflammatory chemokines/cytokines such as Tumor Necrosis Factor (TNF)-α, interleukin (IL)-6 and IL-1 and the production of compounds that limit inflammation and have an immune suppressive effect, such as IL-10 and Transforming Factor (TGF) β. IL-6 and IL-6/soluble IL-6 Receptor (R) complex stimulate liver cells to produce inflammatory proteins, which represents the systemic inflammation response. The magnitude and the duration of the systemic inflammatory response are linked to the cause, under genetic and epigenetic control. Significant inflammation as seen in septic shock, in severe forms of infections or in certain active cancers, represents the “bad inflammation”, correlated with a poor prognosis. In addition, the persistence of a chronic smoldering inflammation may lead to pathological situations which are observed in the majority of inflammatory, degenerative, dysmetabolic, or dysimmune diseases and cancer. Chronic smoldering inflammation is a cross between different pathological situations possibly linked. In addition, within the tumor microenvironment, inflammatory process results from different cellular mechanisms modulated by metabolic and vascular changes. On the contrary, a limited and balanced inflammation initiates the normal immune response, including the adaptive response which amplifies any immunotherapy, including vaccines. Immune checkpoint inhibitors and chimeric antigen receptor (CAR) T-cells are associated with cytokine release syndrome, a clinical risk leading to the use of anti-cytokine drugs. Nowadays, it is time to monitor the dynamic inflammatory process for a better immune precision medicine in both infections and cancer.

## Introduction

Inflammation is an essential and normal biological mechanism for response to an injury in humans. The first historical indications of inflammation, using herbology, was introduced in China by the mythic emperor Shennong, 5,000 years before. Hippocrates recommended extracts of willow bark to limit inflammation conducting to the salicilin identified in 1828 by Johannes Buchner and the chemical synthesis of acetylsalycilic acid by Charles Frédéric Gerhardt in 1853. The first description of the symptoms associated to inflammation, “rubor et tumor cum calore et dolore” was made in the first century by Aulus Cornelius Celsus. In the late 19^th^ century, inflammation associated to infection and the germ theory of disease was particularly introduced by Robert Koch and Louis Pasteur. In the same time, Rudolf Virchow observed infiltrated immune cells where cancer lesions appear in inflammatory tissue. In parallel, cellular aspects of the inflammation process were observed, including neutrophils and macrophages. In 1986, Dvorak defined tumors as “wounds that do not heal” and showed that carcinogenesis and inflammatory conditions have common developmental pathways (1). More recently, the cellular and molecular mechanisms of the local inflammation were identified with a local inflammatory response followed by a regional adaptive immune response and systemic inflammation to amplify the initial biological response.

Inflammation process is a protective response against foreign bodies or injuries, as observed in host tissues. It begins with enhanced vascular permeability of capillaries and leukocyte recruitment through endothelial activity. Normal controlled inflammation leads to healing and specific immune response through the activation of different mechanisms and particularly the antigen presentation with the activation and the maturation of antigen presenting cells. Amplified, uncontrolled, or prolonged inflammatory processes are associated with clinical symptoms and may lead to diseases including cancer. Systemic inflammation is associated to high proliferation rate and poor prognosis in cancer patients. The presence of inflammatory or immune cells within the tumoral microenvironment has been extensively studied particularly in the context of immune therapy aiming to predict clinical response to such treatment. On the contrary, the lack of a normal inflammatory response and/or an amplified immune suppression are also associated with pathological situations. In both sepsis and cancer, biological balance is essential, between a “good inflammatory response” that leads to a specific immune response, and a “bad inflammatory response” which is associated with an immune suppression leading to complications and disease progression. However, inflammatory process is changing over the time, reflecting the degree of the normal or abnormal response of the body. For all of these reasons, the dynamic evaluation of the inflammation process is mandatory in all diseases associated with inflammation. In this review, we discuss the biology of the normal and abnormal inflammatory response, trying to understand both the best analysis and thus, the best therapeutic targeting of such a dynamic by comparing inflammation during infection and cancer.

## Clinical Observations

### “Good inflammation”: The Beginning of a Normal Immune Response

**Figure 1:**
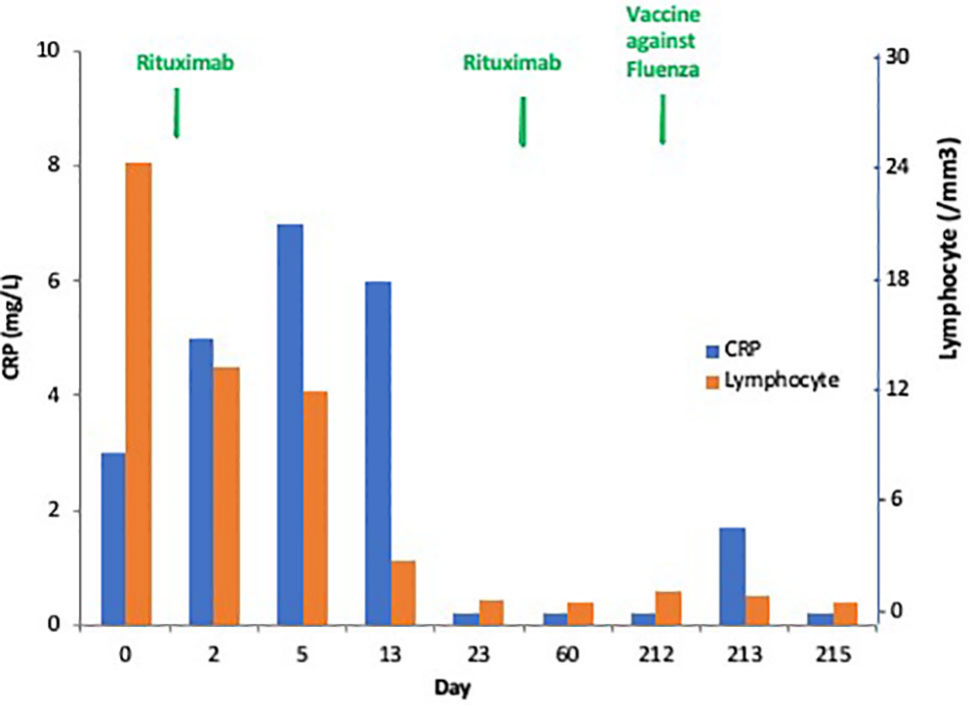
Dynamics of the “good” inflammatory response in a patient with progressive mantle cell lymphoma with leukemic phase and mutated TP53. This patient experienced a complete response after one injection of rituximab followed by a tumor flare syndrome and total disappearance of the leukemic infiltration (no CD19+ cells without mutated TP53 + cells). This patient had a transient C-reactive protein (C-RP) increase in the serum peaking at Day 5, and he is always in complete response at +9 months.

As observed in **Figure 1**, the favorable anticancer immune response observed in a particular patient having mantle cell lymphoma with poor prognosis was associated with a transient and limited inflammatory response. This observation illustrates a good inflammatory response which is the beginning of an adaptive immune response, both at cellular and humoral response levels. This controlled and transient inflammatory response is usually observed after vaccination and correlated to the reactogenicity, as described in hepatitis B and Salmonella typhi vaccines, and as shown also in **Figure 1** (2–4). Different cytokines are associated with antigen presentation and maturation to cooperate with other immune cells (5). The time course and the magnitude of this inflammatory response included serum interleukin (IL)-6 peaking at 12 h after an effective vaccination, ranging from 6 to 8 pg/mL and equally observed after each dose of vaccine. As expected, C-reactive protein (CRP) serum level peaks at day 1–2, ranging from 4 to 9 mg/L and is correlated with the IL-6 peaks (2). Similarly, interferon-γ and different serum levels of chemokines such as interferon γ induced protein (IP)-10, macrophage inflammatory protein (MIP)-1β and monocyte chemoattractant protein (MCP)-2 were higher after the second dose of the most effective hepatitis B vaccine (2).

### “Bad Inflammation” Is Associated to Abnormal Immune Response

A bad inflammation is an inflammatory response deregulated either in its duration or its amplitude. The cytokine storms observed during sepsis or after chimeric antigen receptor (CAR) T-cell therapy are clinical situations associated with life-threatening and represent an excessive inflammatory response (6–8). The presence of a smoldering chronic inflammation, described in the process of aging named inflammaging, as observed with congestive heart failure or chronic pulmonary disease, greatly reduces the specific immune response as demonstrated with varicella-zoster vaccine (9, 10). This low-level of persistent chronic inflammation has been observed to be associated with generalized atherosclerosis and used for cardiovascular risk stratification (11, 12).

### Inflammation and Cancer

Inflammation is a well-known hallmark of cancer. A deregulated inflammatory process could be considered as the cause or consequence of cancer. Infectious diseases and chronic inflammation account for approximately 25% of cancer-causing factors (13). Several infectious agents, including parasites, bacteria, and viruses have been demonstrated to be associated with different cancers through the production of reactive oxygen species (ROS) produced by inflammatory cells as well as epithelial cells, causing DNA damage in addition to epigenetic alterations.

Inflammation is associated with the risk of cancer, but also with the progression or metastatic risks, through different mechanisms such as chronic inflammation, tumor-associated inflammation, therapy-induced inflammation, or epigenetic conditions caused by environmental exposure or nutritional exposure (14). Paraneoplastic syndrome with B symptoms such as fever, sweats, weight-loss, and biological inflammatory symptoms, is correlated with a poor prognosis and advanced disease or metastatic risk (15, 16). In addition, excessive systemic inflammation is associated with immune suppression, thus amplifying the risk of secondary infection or the cancer progression (17).

In mice models, the creation of a local inflammation in the peritoneum leads to the appearance of a plasma cell tumor through the production of IL-6, a survival, and proliferation factor of myeloma cancer cells (18, 19). In plasma cell neoplasia, IL-6 is considered as a paracrine proliferation factor that may become an autocrine factor when the tumor proliferation became independent from the cancer micro-environment (20). IL-6 has been shown to be a survival and/or proliferation factor in certain cancers, such as kidney, ovarian, prostate, and certain types of B-cell lymphopathies, and can therefore be considered a therapeutic target (21). Generally, this very important inflammation is associated with a high proliferation rate of cancer cells which increases chemokine and cytokine production which attract even more immune cells into the cancer microenvironment and lead to tumor cell death and necrosis. The immune microenvironment of the tumor is changing under the pression of different elements, including hypoxia, angiogenesis, tumor mutations, and the production of different chemokines and cytokines in a permanent dialogue between cancer cells and immune surveillance supported by the immune effector cells (22). Hypoxia is associated with a metabolic change of immune cells, from mitochondrial respiration to glycolysis which turns the T-lymphocytes to T-regulator cells (T-reg) which share tolerogenic effects (23). In addition to T-regs, myeloid cells including myeloid derived suppressor cells (MDSC) and neutrophils have been associated with tumor progression and poor prognosis (24, 25). Immune suppression is initiated by the production of a panel of inflammatory cytokines including IL6, IL1, IL10, IL11, and transforming growth factor (TGF) β, mainly produced by macrophages (26). In addition to the changes observed within the cancer microenvironment, circulating MDSC concentrations, neutrophils, and neutrophil:lymphocyte ratio, are correlated with metastatic disease in different cancers such as melanoma, breast, and gastrointestinal cancers (27). The deregulation of different pathways implicated in cancer growth like the loss of tumor suppressors and/or activation of oncogenes influences the formation of an anti-inflammatory microenvironment. Due to the cytokine and chemokine changes, intracellular expression of transcription factors such as NF-κB or STAT3 is activated (28), a situation that is amplified in obesity (29).

## Inflammation Processes

**Figure 2:**
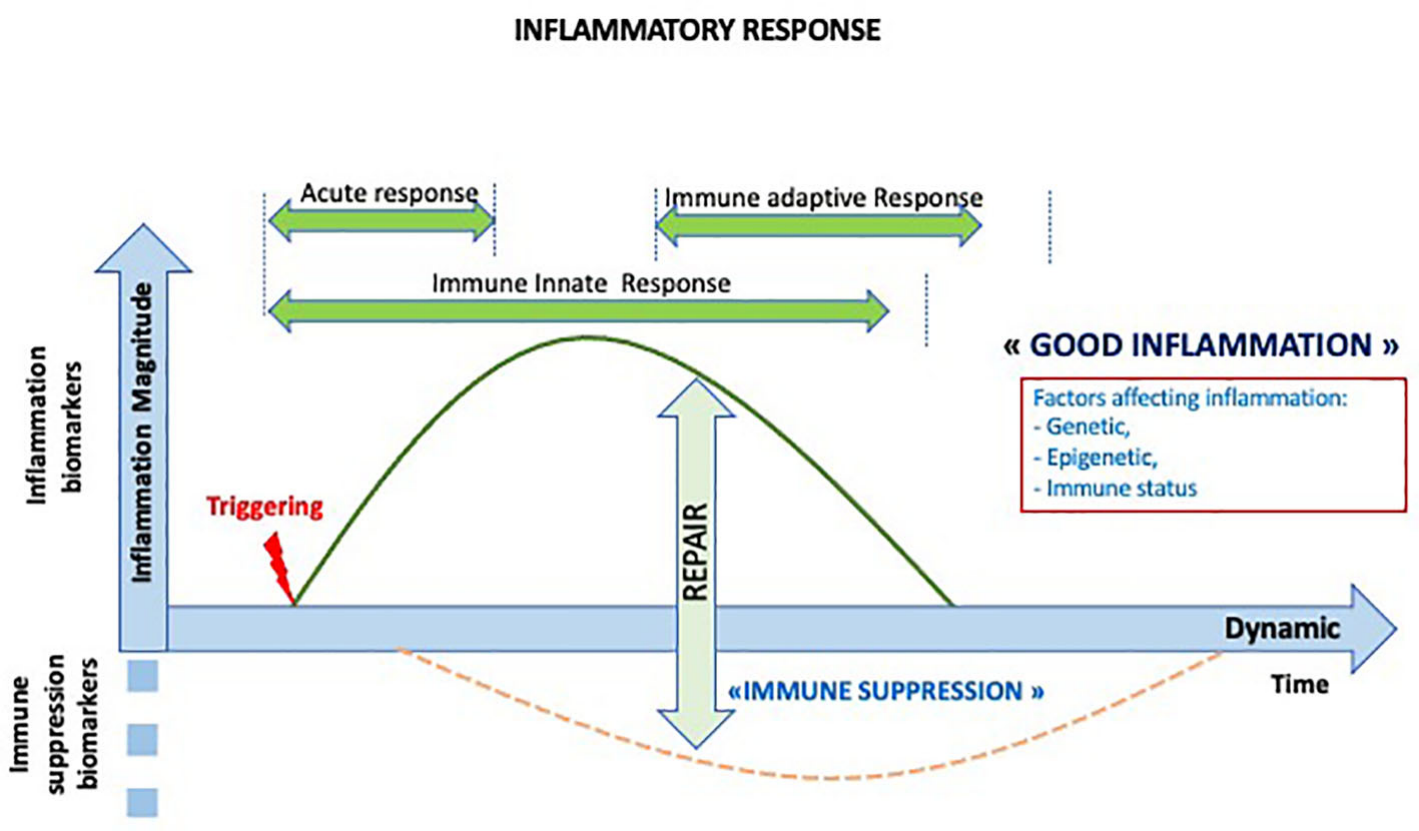
Dynamics of the normal inflammatory response. The inflammatory response is a transient response followed by transient immune suppression and repair period and the initiation of the adaptive immune response.

The onset of a normal acute inflammatory response requires triggering factors, rapidly followed by a repair phase reflecting the transient nature of this process (**Figure 2**). The inflammatory process has been particularly studied in the context of bacterial infection. However, there is a common inflammatory response to any injury, with inducers, sensors, mediators, and effectors (30).

### Inducers

Inducers can be exogenous or endogenous. Exogenous inducers are infectious or non-infectious stimuli and are classified as pathogen-associated molecular patterns (PAMPs) and damage-associated molecular patterns (DAMP) or “alarmins” that are released from damaged cells of the host (31). PAMPs included nucleic acids particularly from viruses or bacteria, proteins such as pilin and flagellin from bacteria, cell wall lipids such as lipopolysaccharide (LPS), lipoteichoic acid from bacteria, or carbohydrates (mannan or glucans) from fungi or bacteria (32). DAMPs are stress-induced proteins such as heat shock proteins (HSP), crystals proteoglycans, mitochondrial components or nuclear proteins such as high mobility group box 1 (HMGB1), several members of the S100 calcium-binding protein family (S100A8, S100A9 and S100A12), histones, and other molecules released when cells are damaged (33). All of these endogenous inducers engage different sensors which are specialized cell-associated recognition molecules of the innate immune system that are activated by the inducers and trigger the production of mediators.

### Sensors

The different endogenous inducers of inflammation obtained after necrotic cell death have also different sensors, such as advanced glycation end-product-specific receptor (RAGE) for HMGB1 and S100A12, purinoceptors (including P2X) for ATP binding resulting in K^+^ ion efflux and cooperate with the NACHT, LRR, and PYD domains containing protein (NALP)3 inflammasome. Such inducers may have crossed or specific activities, like ATP which also activates nociceptors reporting tissue injury to the nervous system, or receptor for RAGE that cooperates with Toll-like receptors (TLRs) (30). There are also soluble of PAMPs, including, pentraxins such as CRP, collectins, ficolins, or complement (C3b) molecules (34, 35). Sensors are expressed by phagocytes, primarily macrophages and neutrophils, dendritic cells (DC), epithelial cells at the barrier of the body, endothelial cells, mast cells, and other types of cells within tissue. Some of these pattern recognition receptors are cell associated including TLRs (1–9), nucleotide oligomerization domain receptors (NOD)-like receptors (NLRs, NOD1-2, inflammasomes), retinoic acid-inducible gene RIG-like receptors (RLRs) (MDA-5), cytosolic DNA sensors (CDSs), C-type lectin-like receptors (CLRs), including mannose receptor, DC-sign, Dectin-1 and 2, scavenger receptors (CD36), N-Formyl met-leu-phe receptors (34).

### Mediators

Inducers of inflammation trigger the production of several inflammatory mediators. Many of these mediators are derived from plasma proteins or secreted by cells, mainly resident mast cells, platelets, basophils or macrophages. These mediators have effects on the vasculature and the recruitment of leukocytes and are classified as vasoactive amines, vasoactive peptides, fragment of complement components, lipid mediators, cytokines, chemokines, and proteolytic enzymes (30). Lipid mediators derived from phosphatidylcholine, included eicosanoids and platelet activating factors. In addition to the promotion of inflammation, mediators also initiated tissue repair after injury. Inflammasomes are cytosolic protein platform assembled in response to danger signals. Inflammasome complexes contain a sensor protein, an adaptor protein and a zymogen, procaspase-1 that is activated into an active enzyme, caspase-1. Activated caspase-1 subsequently activates pro-inflammatory cytokines IL-1β and IL-18, and through the activation of gasdermin D, it induces pyroptosis, a highly pyrogenic inflammatory form of death (34, 36, 37).

After DAMP or PAMP activation, neutrophils are activated directly or indirectly through the production of inflammatory mediators such as (C-X-C motif) ligand (CXCL)-1 and CXCL-2 which binds to and activate G-protein-coupled receptors on neutrophils (38).

### Effectors

**Figure 3:**
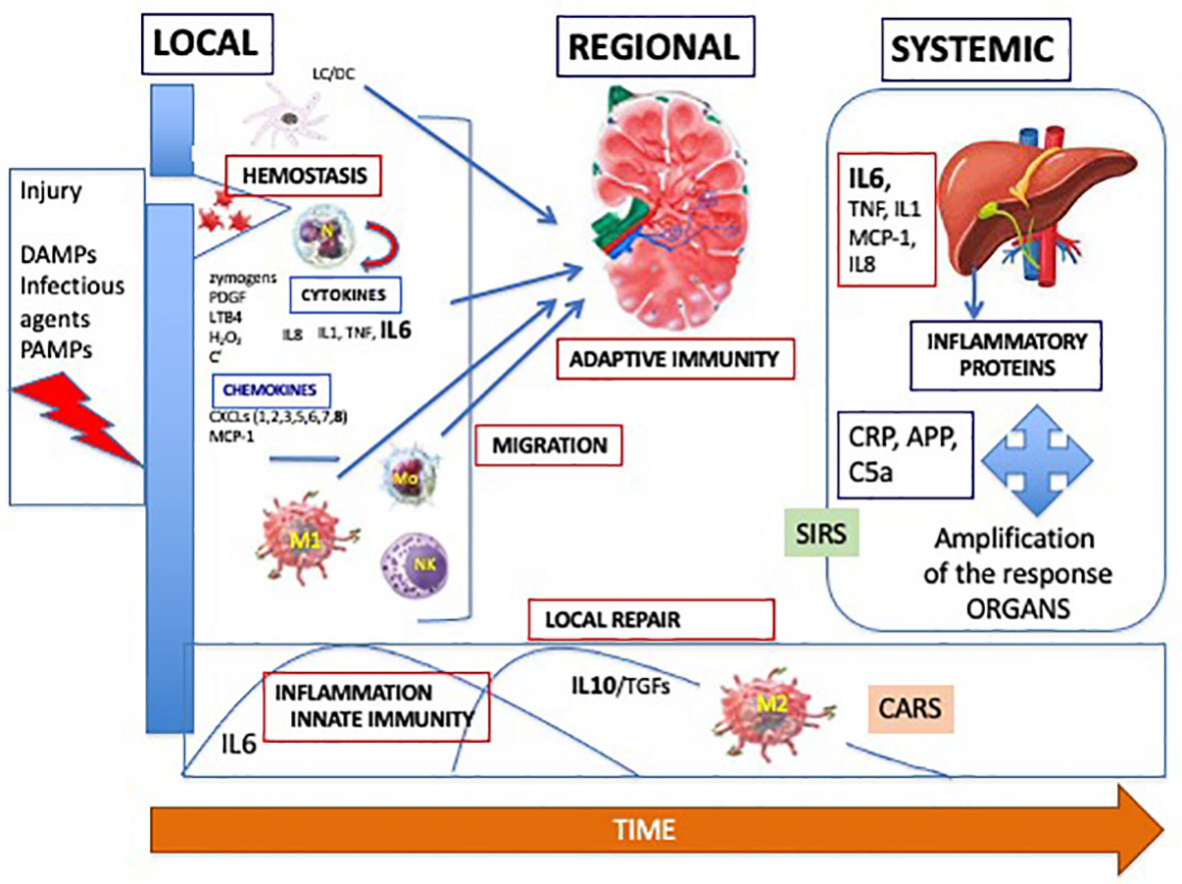
Dynamics of the adapted inflammatory/immune response, with a local, regional in the lymph node and systemic processes. DAMPs, damage-associated molecular patterns; PAMPS, pathogen-associated molecular patterns; PDGF, platelet activating factor; LTB4, leukotriene B4; C, complement; IL, interleukin; TNF, tumor necrosis factor; CXCL, chemokine C-X-C- motif ligand; MCP-1, monocyte chemoattractant protein; M, macrophage; NK, natural killer; Mo, monocyte; TGF, transforming growth factor; CARS, compensatory anti-inflammatory response syndrome; SIRS, systemic inflammatory response syndrome; CRP, C-reactive protein; APP, amyloid protein P.

Effectors are all the cells present or called to the initial site of the injury. They include neutrophils, natural killer (NK) cells, monocytes/macrophages and T- and B lymphocytes. This local response is prolonged and amplified by a regional and a systemic inflammatory/immune response as shown in **Figure 3**.

Neutrophils arrive at sites of damage or infection within minutes after migration along chemotactic agents. Intravascular migration of neutrophils starts by “rolling” on the endothelium of blood vessels which is mediated by selectins and followed by chemokine activation through a conformational change of the G protein coupled receptor leading to activation of neutrophil integrins such as very late antigen (VLA)-4 (CD49D/CD29), macrophage-1 antigen (MAC-1, CD11b/CD18), and lymphocyte function-associated antigen 1 (LFA-1, CD11a/CD18) (39). This integrin activation favors the cell adhesion to endothelium through the Immunoglobulin (Ig)-superfamily cell adhesion molecules (ICAMs). They shed microvesicles that include chemotactic signals such as leukotrienes from arachidonic acid from lipid membranes after inflammasome activation (40). Neutrophils have a relatively short life, dying *via* apoptosis particularly in anti-bacterial activity that include phagocytosis and the production of ROS, proteases, and neutrophil extracellular traps (NETs) (41). NET is a network associating DNA coated with histones, elastase, myeloperoxidase (MPO), and cathepsin G, initially observed in bacterial cell killing and leading to cell death and named lytic NET (42, 43). In addition, it was demonstrated that NET formation can occur independent of cell lysis and subsequent cell death in non-infectious stimuli. In such a non-lytic NET release, this mechanism appears more rapidly in 5 to 60 min as compared to 3 to 5 h for the lytic NET release. This non-lytic NET release is mitochondrial and not nuclear, lacking histones (44). Recently, it was demonstrated that it is not all neutrophils that release NETs, but more particularly the low-density neutrophils, generally CD177 negative and expressing olfactomedin 4 (OLFM4) and which secrete this molecule after the release of NET (45). A recent study has shown that patients with a high percentage of OLFM4+ neutrophils are associated with high risk of septic shock and organ failure (46). In addition, OLFM4 forms complexes with a number of binding molecules included in major signaling pathways such as nuclear factor kappa B (NF-κB) and wingless Int-1 (Wnt), that play main roles in carcinogenesis and inflammation. However, neutrophils are cells difficult to analyze, due to their short life span, their changes after *ex vivo* manipulations. Recent advances, using automated flow cytometers, have permitted to better analyze neutrophil subpopulations based on their size, nuclei aspect, and the presence of different surface biomarkers including CD62L, C5aR, CD11b, FcγRII, associated with inflammatory/immune activation as well as immune suppression (47). MPO is mainly produced by neutrophils and belongs to the family of heme-containing peroxidases. Its activity involves the production of ROS and is released into extracellular fluid after oxidative stress and inflammatory responses (48). Thus, a controlled MPO release at the site of infection is crucial, and abnormal release or function is associated to different diseases expressing chronic inflammation. Cell-cell interactions between neutrophils, endothelial cells, monocytes, and lymphocytes are essential, with the expression of different molecules interaction including integrins.

Neutrophils and monocytes/macrophages arise from common precursors, and co-express similar antigens and produce similar effector molecules including chemokines, cytokines, oxidants (49). However, they have different activities in the inflammation process, particularly in defense against pathogens. Neutrophils are the first cells present in injured tissue, having higher microbiocidal activity, whereas monocytes/macrophages are recruited later on, and they participate to the initiation of adaptive immune response through digestion and antigen presentation. Monocytes are heterogeneous cells that circulate in peripheral blood, representing a central cell family in the context of innate immunity and inflammation. In humans, three subpopulations of monocytes have been described, including CD14++CD16- classical, intermediate CD14+CD16+, nonclassical CD14-CD16++ monocytes (50). Tissue-resident macrophages have major functions in resolution of inflammation and tissue repair (51). Neutrophils are programmed to quickly die to prevent excessive inflammation. Macrophages act to prolong their survival by producing growth factors such as granulocyte-macrophage colony-stimulating factor (GM-CSF), G-CSF, and tumor necrosis factor alpha (TNF-α) (52). Macrophages are able to acquire either the “M1” pro-inflammatory phenotype or the “M2” anti-inflammatory phenotype (53). M1 macrophages express inducible nitric oxide synthetase and CD40. They produce cytokines such as IL-6 and TNF-α and are particularly active in case of infection (49, 54). M2 macrophages express arginase I and CD206 and produce TGF-β and IL-10 to start tissue repair. Neutrophil heterogeneity and plasticity have been recognized and these cells may differentiate into different subsets that interact and modulate macrophage activity. The activity of neutrophils and the polarization of monocytes/macrophages are associated to metabolic changes due to the energy required and REDOX regulation. Neutrophils are more dependent on glycolysis to provide rapid energy necessary to ROS production. Monocytes first use mitochondrial oxidative phosphorylation and glycolysis depending the phase of the immune response, primarily inflammation and then resolution and tissue repair activation (40).

### Inflammation Homeostasy Regulation and Dynamics

The regulation of inflammation takes place in a sequence of initiation, amplification and control of the inflammatory process with a view to repair. The mechanisms present during these different phases bring into play the mediators and cells described previously. The homeostatic regulation maintains the levels of glucose and oxygen concentrations at acceptable range depending the level of inflammation which is extreme in the context of infection and injury, and the lack of any tissue malfunction.

**Figure 4:**
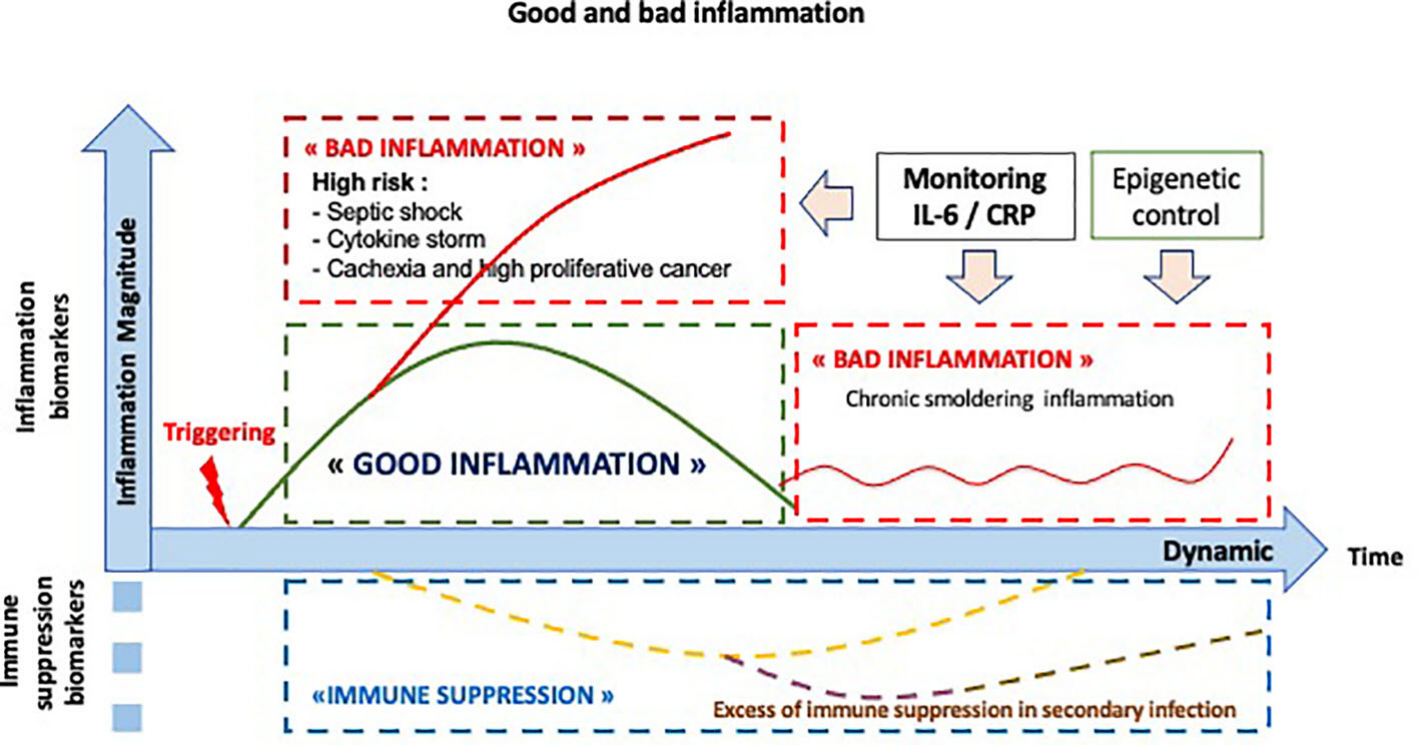
Dynamics: the normal and abnormal inflammatory response. The normal inflammatory/immune response is noted in green. The abnormal inflammatory/immune responses are noted in red, including a high level of inflammation, as observed in septic shock or in cytokine storm, a chronic smoldering inflammation or an excess of immune suppression, named CARS (compensatory anti-inflammatory response syndrome) in infection process.

The inflammatory response is a dynamic controlled response. Thus, the analysis of the pathological inflammatory response cannot be conceived of apart from a dynamic study relating to its amplitude and duration. As shown in **Figure 4**, there are several pathological situations, including the cytokine storm observed during infections, inflammatory cancers generally associated with a poor prognosis or immunoresistance, and persistent chronic inflammation, a risk factor for disease.

The pro-inflammatory response is generally self-limited, even in the absence of effective therapy. However, in some patients who develop sepsis, the response is hyper-amplified, associated to a “systemic inflammatory response syndrome” (SIRS) and leads to a compensatory down regulation of the immune system, named “compensatory anti-inflammatory response syndrome” (CARS) (55).

This dynamic aspect has been observed for CRP on a longer period mainly in the context of infection. The slow decline in CRP after inflammatory process, as well as the peak of CRP serum level are associated with longer survival in older hospitalized patients on a 6-month period (56). However, this dynamic CRP evaluation does not take in account of the early and rapid changes in inflammatory process, the delayed response and the half-life of the molecule, thus reflecting a more complex physiological process.

## Biomarkers of Inflammation

Biomarkers of the inflammatory response are the mediators and effectors of such a process. Usual cites of choice for these biomarkers have centered on their significance with respect to specific diagnostic settings including infection, cancer, trauma, and other pathological situations. The dynamic aspect introduces a three-dimensional vision, time and therefore its duration, amplitude, and speed of its constitution, making it possible to locate the normal or abnormal nature of this inflammatory response as shown in **Figure 4**.

### Effectors

Neutrophil/monocyte lineage and innate cells are early effector cells particularly after an acute injury, such as infection. Analysis of circulating neutrophils, particularly those expressing CD16bright CD62Ldim and described as precursors of MSDC, as well as subpopulations of monocytes were evaluated by flow cytometry for the early detection of infections or after immune suppression (39). Monocyte Human Leucocyte Antigen (HLA)-DR expression decreases rapidly in correlation to the severity and outcome of the septic shock (57). Dynamic changes of monocyte phenotypes have been demonstrated to be associated with severity of the COVID-19 disease, particularly HLA-DR^lo^CD163^hi^ monocytes were present mainly early in severe disease, while HLA-DR^lo^S100A^hi^ monocytes dominated the late phase of disease (58). Recent technological advances have made it possible to better understand the phenomenon of trogocytosis, an essential mechanism of intercellular communication, particularly observed with immune cells in cancer as well as in infection (59, 60).

### Early Mediators

#### Cell Surface Molecules Shedding

Upon leukocyte activation with diverse stimuli, adhesion molecules are rapidly shed from cell surface by proteolytic cleavage. Three of them belonging to the selectin family (E-selectin, L-selectin and P-selectin) and two belonging to the immunoglobulin domain superfamily cell adhesion molecules (intercellular adhesion molecule (ICAM)-1, vascular cell adhesion protein (VCAM)-1), were particularly associated with sepsis (61). The soluble form of cell surface CD14 molecule or presepsin, the receptor of lipopolysaccharide (LPS) is also considered as a biomarker for early infection and its severity and prognosis (62).

#### Chemokines and Cytokines

Among the different cytokines and chemokines produced at the initial phase of the inflammatory process, IL-8, and particularly IL-6 and TNF-α have been extensively studied in inflammatory diseases and infections. They represent the main inflammatory cytokines released in the context of inflammation regardless its cause. IL-6 is a pleiotropic cytokine, with a four-helix bundle structure, having 212 amino acids with a signal peptide of 27 amino acids. Its molecular weight ranges between 21 and 28 kDa, depending on the amount of post‐translational modifications, such as glycosylation and phosphorylation (63). IL-6 is produced by different types of cells, mainly fibroblasts, keratinocytes, monocytes, and macrophages, particularly in early phase of infectious inflammation after stimulation of Toll-like receptors.

In addition to IL-6, monocyte chemotactic protein (MCP)-3 and interferon gamma induced protein (IGP)-10 are early biomarkers of inflammation and have been observed to be excellent predictors for the progression of COVID-19 (64). In bronchoalveolar lavages from patients with severe and critical COVID-19 infection, IL-6 concentration may reach 10,000 pg/mL, 10 to 100-fold higher than that observed in the circulation reflected by the serum CRP concentration. This huge local production of cytokines is probably dependent on both the viral load and the magnitude of the macrophage and neutrophil activation (65).

A normal inflammatory response is followed by the return to a normal situation with immune suppression and repair. Different effectors and mediators are implicated in such a balanced response. Among them, the IL-10 family cytokines are essential to restore the host to an immune quiescent status and tissue homeostasis after inflammatory process. In infection, high levels of serum IL-10 are correlated with mortality in several studies, probably more through the immune suppression status and the risk of secondary infection or in persistent chronic infection particularly observed in mycobacterial or parasite infections (66, 67). In cancer, IL-10 was reported to have paradoxical effects in that both tumor-promoting and tumor-suppressive effects have been observed (68, 69). Thus, dynamic monitoring of serum follow-up of IL-10 in addition to inflammatory proteins and cytokines can provide additional information with regard to the inflammation-immunosuppression balance and its associated risks.

### Secondary Inflammatory Molecules

In physiological conditions, IL-6 modulates the transcription of several hepatic genes, particularly CRP (70, 71) and other acute-phase proteins like serum amyloid A (SAA), α1-antichymoptrypsin, fibrinogen, haptoglobin, and hepcidin that is implicated in inflammatory anemia through the blockade of ferropontin 1, an iron transporter (72–74). On the opposite, IL-6 reduces the level of albumin, transferrin, fibronectin, and cytochrome P450, and more recently it was demonstrated that CYP3A4 mRNA expression was most reduced by IL-6 followed by CYP2C9 and CYP2C19 (75).

CRP has been extensively studied as a biomarker of both acute and chronic inflammation. Elevated CRP/IL6 levels in the serum are a hallmark of inflammatory and aggressive cancers, particularly associated with lung, colon, and ovarian cancers (76, 77). In addition, in ovarian cancer, a meta-analysis suggests that high CRP levels superior to 10 mg/L, rather than circulating pro-inflammatory cytokines might contribute to the etiology of ovarian cancer (77). As CRP has a longer half-life, its dosage may represent an easiest dosage than IL-6 measurement for large cohort analysis particularly for risk evaluation (78). However, as CRP has no specificity, the definition of chronic inflammation necessitates a dynamic view using highly sensitive dosage, outside the context of an acute inflammation. In a recent meta-analysis pooling 83,995 participants from 14 studies, elevated CRP using a highly sensitive test has been shown as an independent predictive biomarker of mortality both for all causes with pooled RR at 1.75 (95 CI 1.55–198) and for cancer mortality, particularly in men, with pooled RR at 1.25 (CI 1.13–138) (79). In the Copenhagen General Population Study with approximately 63,500 individuals, those with a high baseline CRP (>3 mg/L) had an 80% greater risk of early death compared with those with low CRP levels (<1 mg/L) (80). Approximately 70% to 90% of colorectal cancer (CRC) arise from adenomatous polyps and it develops trough a gradual accumulation of genetic and epigenetic changes, increasing with age and chronic inflammation associated with obesity, inflammatory bowel diseases, and dysibiosis. CRP has been associated with a higher risk of CRC in some studies, but this correlation could have some biais, or high levels of CRP just represent the resultant of different causes leading to a chronic inflammation (78).

Questions also arise for the different biomarkers as cause or consequence of a cancer status (81). Different gene polymorphisms of CRP and inflammatory cytokines or biological mechanisms implicated in inflammatory process have been explored with heterogeneous concluding data, depending the type of gene polymorphism and the type of cancer, mainly in colorectal cancer (81). In the Rotterdam study, baseline CRP levels are associated to chronic inflammation preceding lung cancer, even after subtracting a 5-year latent period, as well as a single nucleotide polymorphism of CRP variation of CRP gene (82).

In the context of infection, other biomarkers have been extensively studied, including pancreatic stone protein/regenerating protein (PSP/reg) and procalcitonin (PCT). PSP/reg is a lectin-binding protein that was firstly described in pancreas and was shown to activate polymorphonuclear cells. It is considered as an acute-phase protein regulated by cytokines produced in the damaged tissues. Recently elevated serum levels of PSP/reg have been correlated with the severity of infection, particularly in adults and newborns (83). PCT is a member of the calcitonin gene-related peptides and the precursor of the hormone calcitonin. The produced PCT is stored in the Golgi apparatus, explaining the low concentrations observed in the blood circulation. Many cytokines except interferon γ contributed to the up-regulation of CALC-1 observed in all cells of the organism, resulting in the higher levels in bacterial infections. PCT has a half-life of 22-29 h and the secretion in bacterial infections starts to rise 4 h after the onset of infection, peaking at 12–24 h, earlier than CRP which peaks at 2–3 days with a half-life of 12–24 h (84). The intra- and inter-individual variation of PCT concentration in the peripheral blood limits its interpretation as a single dosage, thus needing serial measurements for a dynamic evaluation (85). Several prohormones, such as adrenomedullin, atrial natriuretic peptide, and arginine vasopressin have been associated with pneumonia (86). Triggering receptor expressed on myeloid cells (TREM)-1) is a glycoprotein member of the Ig family which is upregulated during inflammation linked to infection as well as in non-infectious inflammatory conditions (87). TREM-1 is not detectable in healthy individuals but only measured in body fluids in response to infection (55).

However, CRP is the strongest and easiest biomarker, largely diffused and used but also unspecific in lower dosages (under 90 mg/L). Heterogeneity of the quality of the results is partly due to the technique used in the different studies, mainly plasma (or serum, sometimes), sample collection variable from 4 h to 48 h, variation of the frozen materials, all of these technical points impacting on the IL-6 dosage with short-half life and rapid degradation particularly in the serum. The kinetics of the PCT are between that of IL-6 and that of CRP, mainly associated with bacterial infection. A decrease of PCT has been associated with antibiotic prescription, except for high-are risk patients, such as febrile neutropenia.

Thus, there is no ideal biomarker in acute inflammation/infection. Consequently, there is a need for several biomarkers, including biomarkers for cellular activity, dynamic evaluation and particularly the analysis of the balance between the pro-inflammatory and the anti-inflammatory phases. Systemic inflammation is probably deleterious depending its magnitude and/or its persistence as observed in chronic inflammation.

During septic shock and particularly COVID-19 infection, the cytokine storm can cause clinical deterioration at high risk of death, in particular due to the rapidity of its constitution, as measured by IL-6/CRP (88). The dynamic measurement of IL6/CRP is crucial to identify such a critical period, aiming to rapidly control such a clinical situation, generally by administering anti-IL6 or anti-IL6R monoclonal antibodies. In addition, during anti-IL6 therapy, the measurement of CRP represents the main biomarker for defining the efficacy and in particular through the regression curve of this biomarker. Biological efficacy of such therapies is obtained when the circulating CRP is totally abrogated meaning that the bioactive IL-6 is neutralized (89). The optimal CRP regression curve can be determined, knowing its half-life which is above 19 h, and we develop a mathematical model for optimizing such therapy (submitted for publication).

We have established a highly effective protocol and analysis for extended gate FET electronic measurement of the ELISA test using same sandwich-assay approach (90). This methodology was recently developed to measure the levels of CRP in serum from a drop blood (E-Sana SAS, Paris, France). The detection range was optimized for 10 μg/ml cutoff and detection limit observed at 10 ng/ml (submitted to publication). This new Point-of Care rapid immunoassay application will be multiplexed with other inflammatory proteins such as IL-6, opening the way for dynamic analysis of the inflammatory response as well as to explore new zones of the normal fluctuating inflammation.

## Inflammatory/Immune Precision Therapy

Serum CRP and SAA levels have demonstrated to be correlated with circulating IL-6 concentration, thus representing also the best surrogate biomarkers for monitoring patients receiving anti-IL-6 treatments (21). Dynamic analysis of the inflammatory biomarkers may contribute to a more personalized immune medicine, monitoring both the risks associated to vaccine and the therapies needed, aiming to reduce inflammation by using anti-cytokine targeting IL-1, TNFα, or IL-6, or to amplify an immune response by using immune adjuvants (91, 92). IL-6 was shown to play a major role in the maturation of DC, maintaining them at an immature status which is associated with tolerance (93). During the early steps of an infection process, the inflammatory response initiates and amplifies the immune response including DC maturation. At a later stage, newly formed immature DCs are locally induced by an immunological scare left-over by inflammation to amplify tolerance (94).

Under therapy such as immune checkpoint inhibitors, anticancer vaccines or combined therapies, systemic inflammation status at baseline predicts the outcome of the patients (95). However, it is difficult to define the prognostic value of IL-6/CRP in these different studies, due to the variability of the methodology used, either below the median or with different subgroups of values (85–87). Finally, it is clear that the lower CRP levels were associated with a better prognosis (96–98). IL-6 mediated STAT3 activation in the tumor microenvironment inhibits functional maturation of DC to activate effector T-lymphocytes, blocking the anticancer immunity leading to a therapeutic blockade of the IL-6 complex (98). IL-6 signaling has been shown to have both pro- and anti-inflammatory activity, first directed to macrophages and T-and B-lymphocytes differentiation and then promoting the resolution of inflammation (99). The usual cis-signaling is restricted to cells having the IL-6Rα, including liver cells and hemopoietic cells. IL-6 trans-signaling represents the circulating complex IL-6/IL-6Rα binding to the ubiquitous membrane gp130 signal transducer, and it has been shown to contribute to anti-tumor adaptive immunity by guiding lymphocyte trafficking to lymph nodes and tumors. So, it appears important to modulate more precisely the inflammation process, particularly in the context of immunotherapy, and not only to limit the toxicity. The use of anti-IL-6 or anti-IL-6R therapies necessitates reviewing the use of these drugs with the search for an immune modulation through precise and dynamic biological monitoring, including the soluble forms of IL-6 receptors as well as the different conformational changes of CRP, monomeric, and pentameric (100).

In addition, anticancer vaccine administration is followed by a significant but transient increase of CRP/IL-6 peaking at 24h (101). Regarding the origin of these circulating proteins, it has been demonstrated that the serum levels of IL-6 originate, at least in part, from the vaccine site (101, 102). Correlation between inflammation level and clinical outcome under immune therapies are controversial with both positive and negative correlation (102, 103). These contradictory data are due to the lack dynamic analysis of inflammation.

**Figure 5:**
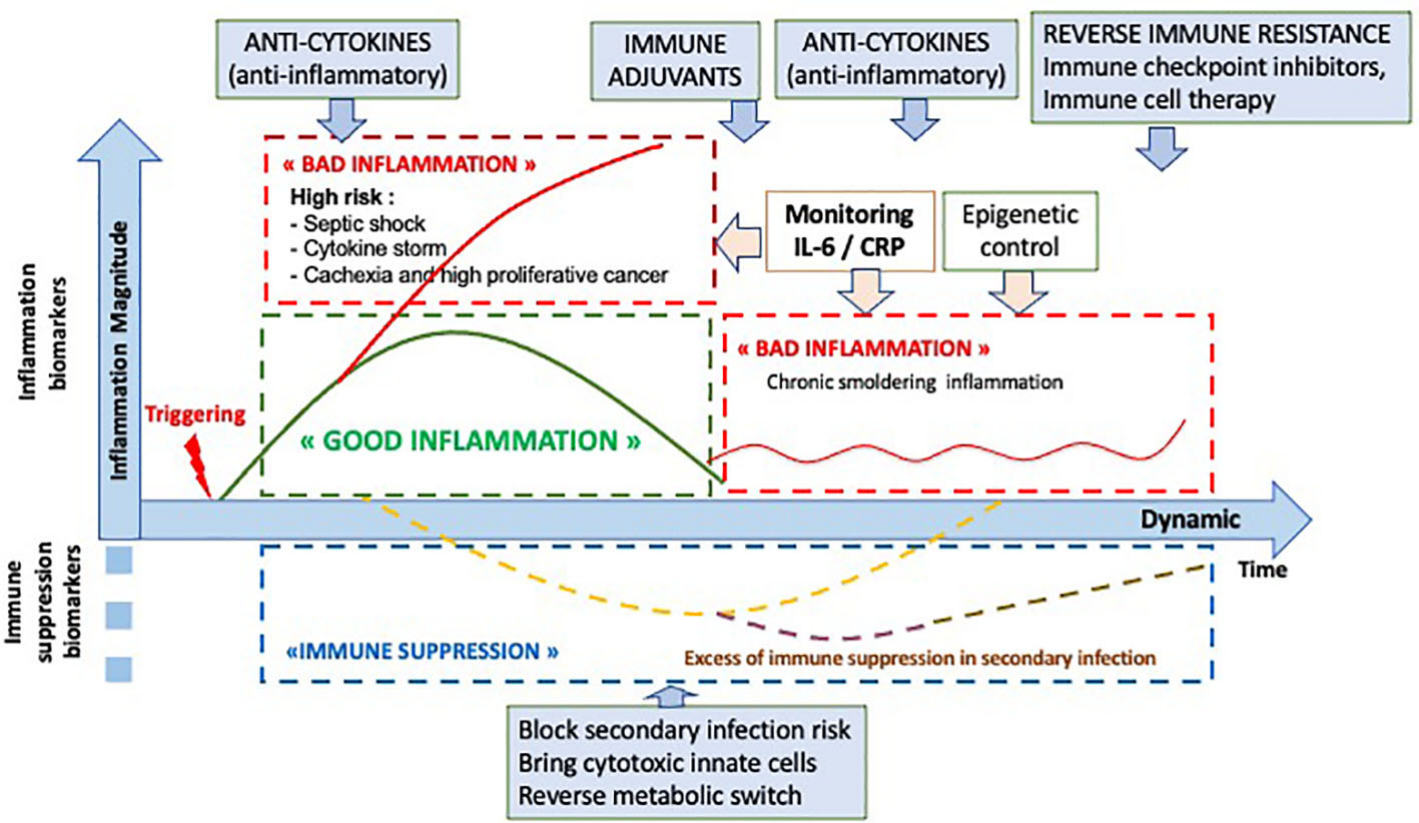
Optional therapeutic interventions depending the dynamic analysis of the inflammatory and immune response.

In conclusion, the inflammatory response is a normal mechanism to initiate the immune response. Its prognostic impact is known, in particular in the context of an excessive inflammatory response such as cytokine storm, as observed during infections or after CAR T-cell therapy. However, the impact of a good inflammatory response requires dynamic monitoring to observe its time course, amplitude and duration. This dynamic vision will thus make possible to resolve interpretation contradictions, opening the way to a new immune precision medicine both in cancer and infections as shown on **Figure 5**.

## Author Contributions

J-FR and ZL made the concept and wrote the article. KL and CM discussed the article and participated to the discussion around technical development. All authors contributed to the article and approved the submitted version.

## Conflict of Interest

J-FR, KL, and CM are co-founders of E-Sana. J-FR is consultant for Leo Pharma, NPO Petrovax and Eusapharma.

The remaining author declares that the research was conducted in the absence of any commercial or financial relationships that could be construed as a potential conflict of interest.
